# Acetylation stabilises calmodulin‐regulated calcium signalling

**DOI:** 10.1002/1873-3468.14304

**Published:** 2022-02-07

**Authors:** Karen Baker, Michael A. Geeves, Daniel P. Mulvihill

**Affiliations:** ^1^ School of Biosciences University of Kent Canterbury UK

**Keywords:** acetylation, calmodulin, endocytosis, myosin, *Schizosaccharomyces pombe*

## Abstract

Calmodulin is a conserved calcium signalling protein that regulates a wide range of cellular functions. Amino‐terminal acetylation is a ubiquitous post‐translational modification that affects the majority of human proteins, to stabilise structure, as well as regulate function and proteolytic degradation. Here, we present data on the impact of amino‐terminal acetylation upon structure and calcium signalling function of fission yeast calmodulin. We show that NatA‐dependent acetylation stabilises the helical structure of the *Schizosaccharomyces pombe* calmodulin, impacting its ability to associate with myosin at endocytic foci. We go on to show that this conserved modification impacts both the calcium‐binding capacity of yeast and human calmodulins. These findings have significant implications for research undertaken into this highly conserved essential protein.

## Graphical abstract

This study reveals that amino‐terminal acetylation of calmodulin is necessary for this essential eukaryotic calcium signalling protein to fold and function correctly. We show this conserved modification impacts the calcium‐binding capacity of yeast and human calmodulins. These findings have significant implications for research undertaken into this highly conserved essential protein.

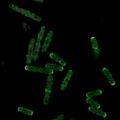

## Abbreviations

**CaM**, calmodulin

**FRET**, fluorescence resonance energy transfer

**hCaM**, human calmodulin

**IAANS**, 2‐(4′‐(iodoacetamido) anilino naphthalene‐6‐sulfonic acid

**NAT**, amino‐α‐acetyl‐transferase

**Nt**, amino‐terminal

Most molecular processes within living cells are controlled by signalling pathways, with signals typically conveyed *via* post‐translational modifications or cation binding. Calmodulin (CaM) is a conserved calcium‐binding protein found in all eukaryote cells to date [1], which is capable of binding 4 Ca^2+^ ions, *via* highly conserved EF‐hand motifs. Association of these divalent cations results in a large conformational change of the CaM (or CaM like protein) [2] to modulate binding to ligand proteins and regulate their function. Thus, CaMs can act as signal transducers for many different cellular processes including gene expression, protein synthesis, cell growth, division and muscle contraction [1, 3, 4]. One key class of CaM target protein are myosins, actin‐associated motor proteins, the function of which is regulated by association of CaM light chains to affect motor activity and stability of the lever arm [5, 6].

The fission yeast, *Schizosaccharomyces pombe*, contains two CaM homologues, Cam1 and Cam2. Cam1 is an essential protein, which localises to the spindle pole body and sites of endocytosis (also called actin patches). The conformation of Cam1 is regulated by calcium binding to modulate its association to IQ motifs within ligand proteins, such as myosin motors [7, 8, 9, 10]. This association between Cam1 and these actin‐associated myosin motors plays a critical role in regulating diverse cellular processes within the yeast, including cell division and endocytosis. In contrast, Cam2 is not only a non‐Ca^2+^ binding CaM homologue, but is also non‐essential to the viability of the cell, playing subtle roles in modulating polarised growth in response to changes in the cellular environment [11, 12]. Each CaM associates with the neck region of the class I myosin, Myo1 [10, 11, 13], stiffening the lever arm to regulate Myo1 dynamics during endocytosis [11].

Amino‐terminal (Nt) acetylation is a ubiquitous post‐translational modification, affecting up to 90% of eukaryote proteins [14] to inhibit Nt‐proteolysis, as well as enhancing the structure and function of a range of proteins. This in turn impacts many cellular processes including cell cycle progression, protein degradation and cytoskeletal organisation. Nt‐acetylation is undertaken by a group of amino‐α‐acetyl‐transferase (NAT) complexes, each of which catalyse the addition of an acetyl group to the processed amino‐terminal residue of a polypeptide. Each NAT complex (NatA, NatB, etc.) specifically recognises, interacts with and modifies specific Nt di‐peptide sequences of the elongating polypeptide [15]. As is the case for the majority of NAT complexes, NatA consist of a catalytic and regulatory subunit, Naa10 and Naa15, which, upon cleavage of the initial methionine, acetylate subsequent amino terminal ‐Ala‐, ‐Thr‐, ‐Ser‐, ‐Val‐ or ‐Gly‐ residues of proteins. These terminal residues correlate with the amino termini of CaMs from diverse organisms, indicating them to be Nat A substrates.

We have investigated the impact Nt‐acetylation has upon the structure and function of the essential fission yeast CaM, Cam1. Using live cell imaging we show that NatA‐dependent acetylation of Cam1 specifically impacts its endocytic function. Using biochemical analysis of recombinant bacterially expressed amino‐terminally acetylated Cam1, we show this post‐translational modification impacts the helical structure and thermal stability of the Cam1 protein, enhancing its sensitivity to Ca^2+^ and affinity for its major cellular binding partner, Myo1. Finally, we provide evidence that the effect upon calcium sensitivity extends to human CaM, which has implications to understanding universal CaM regulation and function, as well as interpretation of biochemical studies using recombinant CaM.

## Materials and methods

### Molecular biology

The *naa15^+^* gene corresponds to the designated coding sequence *SPCC338.07c* within the *S. pombe* genome. The *naa15::kanMX6 s*train was created as described previously [16] using appropriate templates and primers. cDNA of human *CALM1 (HGNC: 1442)* (kind gift of Kati Torok) was amplified by PCR as an *Nde1 – BamH1* fragment, sequenced and subsequently cloned into the rhamnose‐inducible pET3a (Novagen, Gibbstown, NJ, USA) based vector pRham [17] to generate *pRham‐CALM1*. *S. pombe cam1^+^ (SPAC3A12.14)* and *cam1‐T6C* bacterial expression constructs have been described previously [11].

### Cell culture

The yeast strains used in the study were h^−^ *cam1.gfp:kanMX6 naa15^+^ myo52‐tdTomato:hphMX6* and h^−^ *cam1.gfp:kanMX6 naa15::kanMX6 myo52^+^*. Cell culture and maintenance of these prototroph strains were carried out according to [18] using Edinburgh minimal medium with Glutamic acid nitrogen source (EMMG). All cells were maintained as early to mid‐log phase cultures for 48 h before being used for analyses.

### Protein expression and purification

Unacetylated forms of recombinant proteins were expressed and purified from BL21 DE3 *Escherichia coli* cells, while Nt‐acetylated forms of CaM proteins were expressed and isolated from BL21 DE3 pNatA cells [17]. All proteins were isolated as described previously [11], and both identity and acetylation efficiency were confirmed by electrospray mass‐spectroscopy. Cam1.T6C proteins were conjugated to the cysteine‐reactive synthetic fluorophore 2‐(4′‐(iodoacetamido) anilino naphthalene‐6‐sulfonic acid (IAANS)) as described previously [11]. Each protein was subjected to mass spectroscopic, SDS/PAGE and spectrophotometric analyses to determine mass, purity and protein concentration, respectively.

### Fluorescence spectra

Emission spectra were obtained using a Varian Cary Eclipse Fluorescence Spectrophotometer (Agilent Technologies, Santa Clara, CA, USA) and 100‐µL Quartz cuvette. For fluorescence resonance energy transfer (FRET) measurements samples were excited at 435 nm (CyPet excitation) and emission was monitored from 450 to 600 nm with both slits set to 1 nm. Affinity experiments were carried out using 1 µm of FRET fusion protein, in which the CyPet and YPet FRET pair were separated by both Myo1 IQ motifs, with varying concentrations of Cam1 in a final volume of 100 µL in analysis buffer of 140 mm of KCl, 2 mm of MgCl_2_, 20 mm of MOPS, pH 7.0 with 2 mm of EGTA, CaCl_2_ or Ca^2+^‐EGTA as required.

### pCa determination

One micromolar of Cam1‐IAANS and ^ACE^Cam1‐IAANS were prepared in 140 mm of KCl, 20 mm of MOPS, pH 7.0 buffer, containing 2 mm of EGTA/Ca‐EGTA added as appropriate for each pCa condition. IAANS fluorescence values were plotted at each pCa condition and fitted to a Hill Equation to determine the pCa_50_ value.

### Fast reaction kinetics

Data were collected on a HiTech stopped flow system. Fluorescence was excited at 333 nm using a Hg lamp and monochromator and the fluorescence signal collected at 90° through a 455 nm filter. CaM at 4 µm (all concentrations were final after mixing) was preincubated in 140 mm of KCl, 20 mm of MOPS, pH 7.0 buffer with 25 µm of Ca^2+^ and then rapidly mixed with 75 µm of Quin‐2 (Sigma‐Aldrich, St. Louis, MI, USA). Data were analysed by fitting with a one, two or three exponential function as required using the hitech kinetassist software (TgK Scientific Ltd, Bradford‐on‐Avon, UK).

### Circular dichroism

Measurements were made in 1‐mm quartz cuvettes using a Jasco 715 spectropolarimeter (Jasco UK Ltd, Dunmow, UK). CaM proteins were diluted in CD buffer (10 mm of Potassium phosphate, 500 mm of NaCl, 5 mm of MgCl_2_ pH 7.0) to a concentration of 0.4 mg·mL^−1^. Thermal unfolding data were obtained by monitoring the CD signal at 222 nm with a heating rate of 1 °C·min^−1^. At completion of the melting‐curve, the sample was cooled at a rate of 20 °C·min^−1^. CD data are presented as differential absorption (∆A).

### Live cell imaging

**Fig. 1:**
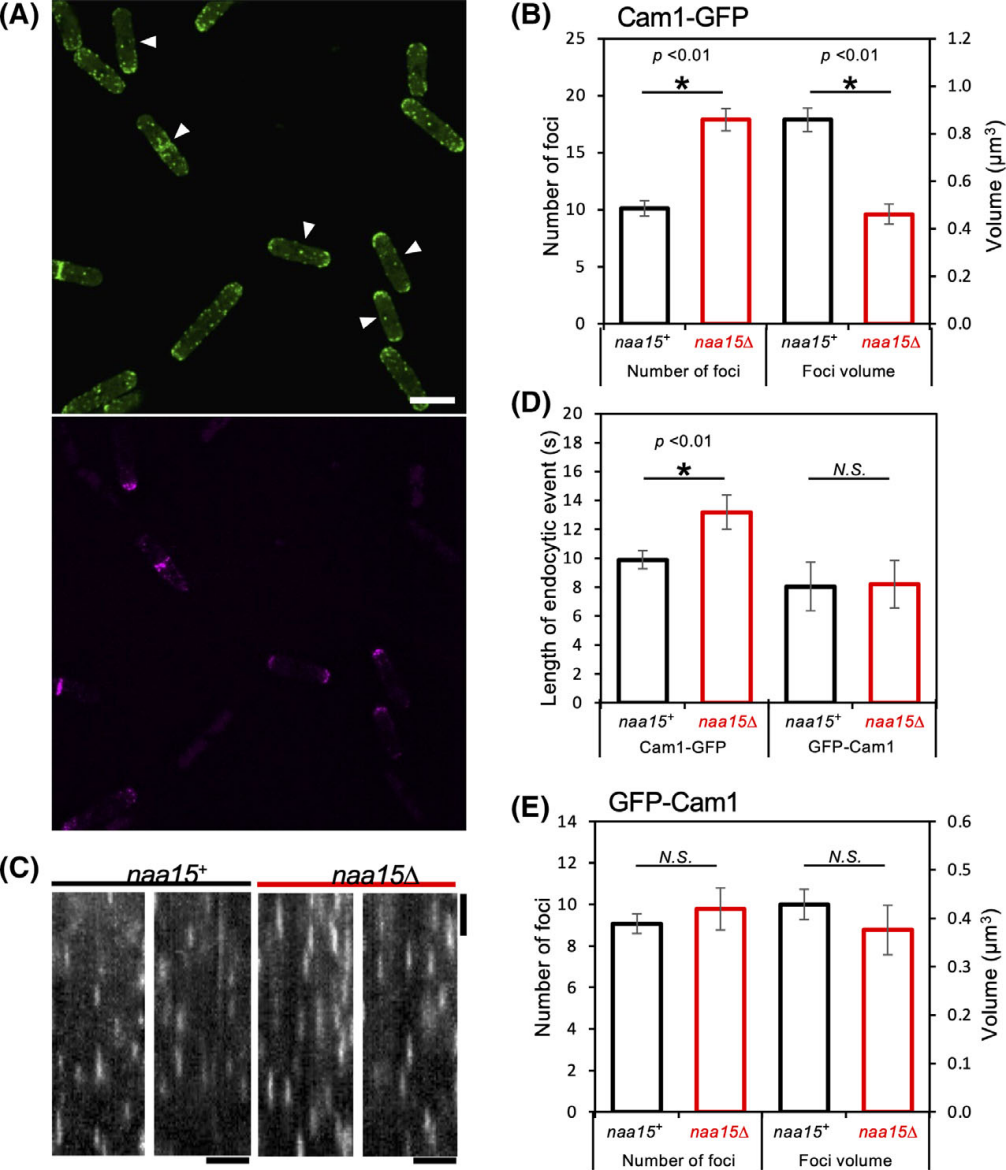
Cam1 distribution and dynamics are disrupted in *naa15∆* cells. (A) Maximum projection of 31 *z*‐plane wide‐field image of Cam1 (green) and Myosin V (magenta) in *cam1‐gfp myo52‐tdTomato* (indicated with arrows) and *cam1‐gfp naa15∆* cells (Scale bar – 10 µm). (B) Analysis of Cam1‐GFP foci automatically detected from maximum projections of 31 *z*‐plane wide‐field images. (C) Typical kymographs of GFP‐labelled Cam1 foci generated from single *z*‐plane time‐lapse images of *cam1‐gfp naa15^+^* and *cam1‐gfp naa15∆* cells (Horizontal scale – 5 µm, Vertical scale – 10 s). (D) Quantification of Cam1‐GFP and GFP‐Cam1 endocytic foci from > 30 kymographs. (E) Analysis of GFP‐Cam1 foci automatically detected from maximum projections of 31 *z*‐plane wide‐field images.

Samples were visualised using an Olympus IX71 microscope with PlanApo 100x OTIRFM‐SP 1.45 NA lens mounted on a PIFOC *z*‐axis focus drive (Physik Instrumente, Karlsruhe, Germany), and illuminated using LED light sources (Cairn Research Ltd, Faversham, UK) with appropriate filters (Chroma, Bellows Falls, VT, USA). Samples were visualised using a QuantEM (Photometrics, Photometrics, Tuscon, AZ, USA) EMCCD camera, and the system was controlled with metamorph software (Molecular Devices, San Jose, CA, USA). During live‐cell imaging, cells were cultured in Edinburgh minimal media using 20 mm l‐Glutamic acid as a nitrogen source (EMMG). Cells were grown exponentially at 25 °C for 48 h before being mounted (without centrifugation) onto lectin (Sigma, St. Louis, MI, USA L2380; 1 mg·mL^−1^)‐coated coverslips with an a Bioptechs FCS2 (Bioptechs, Butler, PA, USA), fitted onto an ASI motorised stage (ASI, Eugene, OR, USA) on the above system, with the sample holder, objective lens and environmental chamber held at the required temperature. Each 3D‐maximum projection of volume data was calculated from 21 *z*‐plane images, each 0.2 µm apart, and analysed using metamorph and autoquant x software (Mediacy Cybernetics, Rockville, MD, USA). Average size and number and cellular distribution of foci were calculated from all foci present within ≥ 30 cells for each sample examined. Timing of foci events were calculated from kymographs (Fig. 1C). The length of the discrete lines in this image correlate precisely to the duration of the Cam1 residence at the foci (1 pixel = 0.8 s).

## Results

### Cam1 amino‐terminal acetylation affects calmodulin organisation and dynamics *in vivo*

Deletion of the NatA regulatory subunit Naa15 in cells, abolishes function of the NatA complex, and therefore NatA substrates remain unacetylated in *naa15∆* yeast [14]. The N‐terminal amino acid sequence of CaMs possess a predicted NatA Nt‐acetylation consensus sequence, which is consistent with proteomics analyses that have shown a proportion (40%) of yeast CAM1 is acetylated *in vivo*, in a NatA complex‐dependent manner [14]. To explore the impact Nt‐acetylation had upon the organisation and dynamics of this essential regulatory protein Cam1‐GFP fluorescence intensity and distribution was examined simultaneously in both *naa15∆ cam1‐gfp* and *naa15^+^ cam1‐gfp myo52‐tdTomato S. pombe* cells, which had been mounted together onto the same coverslip, to allow simultaneous observation of the two strains (Fig. 1A).

While there was no significant difference in cell size and morphology (Table 1), comparison of ^ACE^Cam1‐GFP (Nt‐acetylated protein in *naa15+* cells) and Cam1‐GFP (non‐acetylated protein in *naa15∆* cells) foci within the yeast cell revealed a significant impact upon the *in vivo* distribution of Cam1. A ~ 2‐fold increase in the number of Cam1‐GFP foci was detected in *naa15∆* cells compared to *naa15^+^* (Fig. 1B). In addition, Cam1‐GFP foci are on average 2‐fold smaller than ^ACE^Cam1‐GFP (Fig. 1B). Together, these results are consistent with the total levels of Cam1 observed in *naa15^+^* and *naa15∆* strains (Table 1). Kymographs generated from time‐lapse images (Fig. 1C) revealed Cam1‐GFP remains associated with endocytic patches for significantly longer in *naa15∆* cells (13.2 ± 0.5 s) when compared to equivalent *naa15^+^* cells (9.9 ± 0.4 s) (Fig. 1D).

**Table 1:** Cellular distribution of Cam1 foci.

	cam1.gfp naa15^+^	cam1.gfp naa15∆	*P* value	gfp.cam1 naa15^+^	gfp.cam1 naa15∆	*P* value
Whole cell fluorescence (AU)	31 240 148	34 242 443	0.1968	7 351 958	7 041 604	0.5340
Cell size (µm^2^)	85.0	101.6	0.2142	91.8	106.1	0.1391
Maximum intensity (AU)	127 138	105 098	0.0073	15 802	14 534	0.3806
Number of foci/cell	10.1	17.9	0.0001	9.1	9.8	0.4673
Average foci volume (µm^3^)	0.86	0.46	0.0001	0.43	0.38	0.3612
Total foci volume (µm^3^)	8.38	8.08	0.7586	3.84	3.87	0.967
Total foci fluorescence (AU)	354 818	324 245	0.5048	57 034	59 454	0.6865
*n*	32	36		28	34	

Significant at >99% level of confidence (red).

Comparing the distribution of Cam1 within *naa15+* and *naa15∆* cells reveal the strongly polarised distribution of Cam1 is lost in the absence of acetylation (Fig. 1A). The majority of cellular Cam1 recruits to endocytic patches, which concentrate at sites of polarised cell growth appearing as a cap at the cell tips in wild‐type cells (Fig. 1A). However, analysis of the cellular distribution of > 300 foci across > 30 cells reveals a significant reduction in Cam1 accumulation at cell tips in *naa15∆* cells (77.4% of Cam1‐GFP fluorescence is at the cell tips of *naa15∆ cells compared to* 91.2% in *naa15^+^* cells).

An amino‐terminal GFP‐Cam1 fusion would negate the impact of the *naa15∆* upon Cam1 dynamics. To confirm whether the differences observed in calmodulin dynamics is specifically due to Nt‐acetylation of Cam1 alone, or a consequence of Nt‐acetylation of other proteins, equivalent comparative analyses were undertaken between *naa15^+^* and *naa15∆* cells expressing GFP‐Cam1 [8]. In contrast to the carboxyl fusion, analysis of GFP‐Cam1 distribution in *naa15^+^* and *naa15∆* cells revealed no significant differences in localisation or dynamics between the two strains. Overall fluorescence (Table 1), the number and size of foci (Fig. 1E), and the length of time GFP‐Cam1 associated with endocytic patches (Fig. 1D) were unaffected by the absence of Nt‐acetylation. Thus, the disruption on CaM dynamics and endocytic function observed in *naa15∆* cells is specifically due to lack of Nt‐acetylation of Cam1.

### Expression of N‐terminal acetylated *S. pombe* calmodulin, Cam1

To further understand the mechanism by which acetylation regulates calmodulin function, we carried out *in vitro* biochemical analysis. Although Nt‐acetylation does occur in bacteria, it does so to a significantly lesser extent when compared to eukaryotes [19]. Standard recombinant protein production methods are unable to incorporate eukaryotic NAT complex‐dependent acetyl groups. However, efficient *E*. *coli* expression systems can produce Nt‐acetylated target proteins by co‐expressing NAT complexes [17, 20, 22]. Recombinant calmodulins were produced using a bacterial NatA Nt‐acetylation system [17], which uses the sequential induction of the fission yeast NatA components, Naa10 and Naa15 followed by CaM (Fig. S1A). The accumulation of NatA complex prior to CaM induction ensures efficient post‐translational acetylation of the CaM substrate. Acetylation efficiency was determined by mass spectroscopy (Fig. S1B). Absence of a peak corresponding to unacetylated calmodulin in these samples indicates 100% acetylation efficiency.

### N‐terminal acetylation affects the stability of Cam1 *in vitro*

**Fig. 2:**
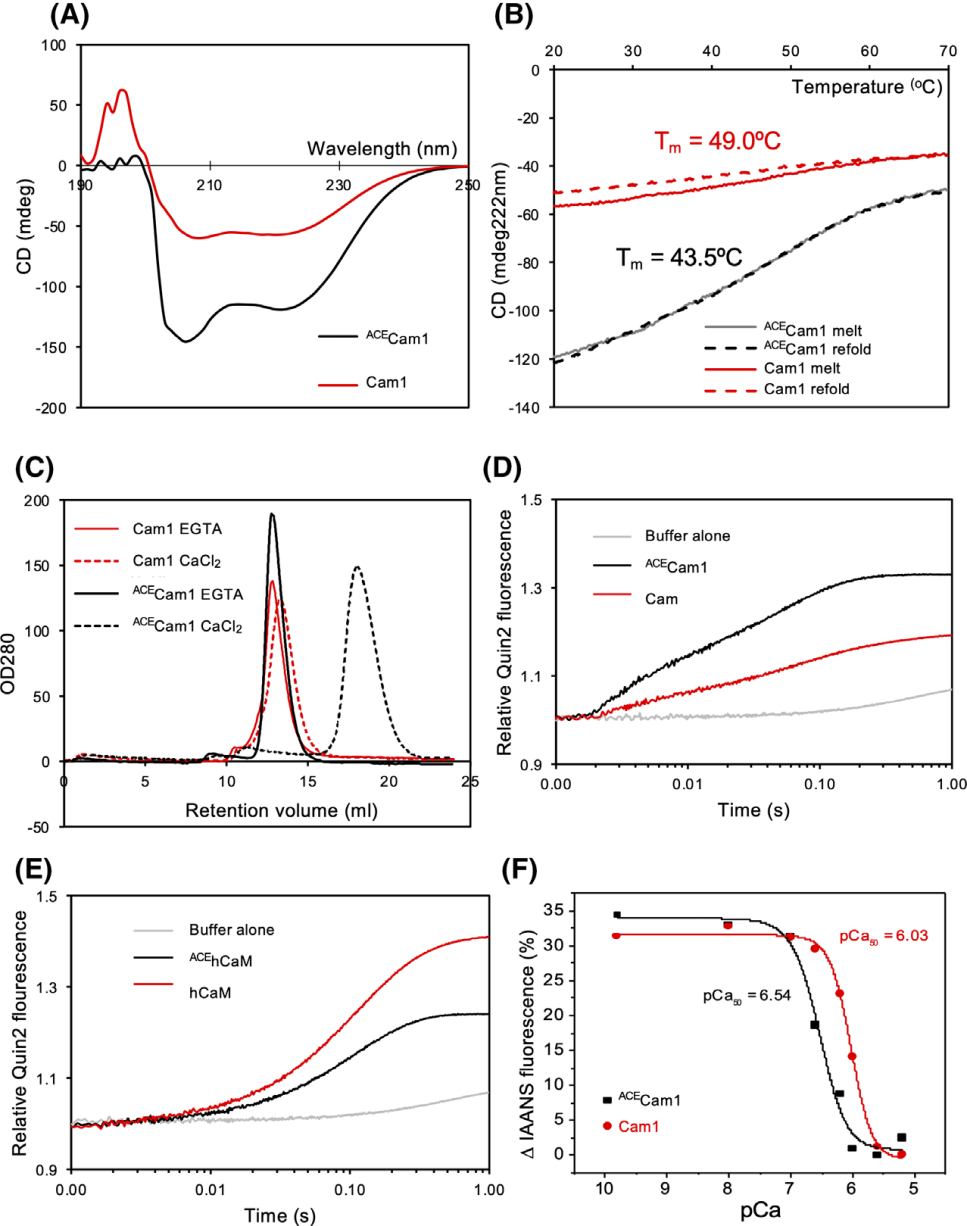
Impact of amino‐terminal acetylated upon Cam1 structure, stability and Ca^2+^ binding. (A) CD spectra of equivalent concentrations of Cam1 (red line) and ^ACE^Cam1 (black line) protein. (B) CD melting curves for Cam1 and ^ACE^Cam1 monitoring CD signal at 222 nm as sample temperature increased to 70 °C (Solid lines) and then returned to 20 °C (Dashed lines). Calculated midpoint melting temperatures (*T*_m_) are shown for both samples. (C) Size exclusion chromatography elution profiles of 100 µm of ^ACE^Cam1 (black lines) and Cam1 (red lines) in the absence of calcium (solid lines) and the presence of calcium (dashed lines). Quin‐2 calcium dissociation experiments from (D) Cam1 and ^ACE^Cam1, and (E) hCaM and ^ACE^hCaM. (F) pCa curves plotting Ca^2+^ dependent changes in IAANS fluorescence of Cam1‐IAANS (red) and ^ACE^Cam1‐IAANS (black) proteins.

The consequence of Nt‐acetylation upon structure and binding characteristics of Cam1 were compared *in vitro*. Circular dichroism (CD) spectra of equivalent quantities of (unacetylated) Cam1 and ^ACE^Cam1 were collected in the absence of calcium (Fig. 2A) to examine secondary structure of the proteins. Both forms of Cam1 had negative peaks at 208 and 222 nm, characteristic of proteins consisting primarily of α‐helices, which is consistent with published structures of calmodulin proteins [23]. However, ^ACE^Cam1 had a lower 222/208 nm ratio (Cam1: 0.91, ^ACE^Cam1: 0.84) suggesting Nt‐acetylation alters the overall secondary structure of Cam1 [24] by stabilising the N‐terminal α‐helix region of the protein, as has been observed in other examples of Nt‐acetylation [25].

CD melting curves revealed Nt‐acetylation affects the thermal stability of Cam1. The α‐helical associated negative peak at 222 nm was followed for Cam1 and ^ACE^Cam1 as temperature was increased from 20 °C to 70 °C, and subsequent re‐cooling and re‐melting (Fig. 2B). Neither form of Cam1 was fully unfolded at 70 °C owing to the high thermodynamic stability of Cam1 [26]. The mid‐point melting temperature for unacetylated Cam1 was 49.0 °C, compared to a lower temperature of 43.5 °C for ^ACE^Cam1. This indicates that Nt‐acetylation increases the thermal sensitivity of Cam1. Despite a reduced unfolding temperature, the refolding curve of ^ACE^Cam1 indicates that all of the protein refolds (Fig. 2B). In contrast, a proportion of unacetylated Cam1 undergoes irreversible unfolding at higher temperatures, consistent with Nt‐acetylation being important for maintaining the structure and long‐term stability of Cam1.

### N‐terminal acetylation affects the calcium sensitivity of calmodulin

A primary function of calmodulin is to facilitate calcium signalling in the cell by regulating the conformation and subsequent function of diverse ligand proteins. Calcium ions bind CaM at 4 EF‐hand domains to induce a major conformational change, thereby modulating affinity to cellular binding partners to have a functional consequence to the cell [27]. Consistent with this ^ACE^Cam1 had differential migration through a size exclusion column in the presence and absence of calcium (Fig. 2C). In contrast, unacetylated Cam1 eluted from the column at similar volume fractions in both conditions (Fig. 2C), indicating that while the unmodified protein was able to bind calcium, it failed to undergo the same conformational change, which is consistent with the CD data (Fig. 2A,B).

To determine whether this difference in conformation was brought about by a failure of the unacetylated Cam1 to bind calcium, we monitored changes in the fluorescence of the Ca^2+^ indicator, Quin‐2 [28] as it displaced Ca^2+^ from CaM. Both Cam1‐ and ^ACE^Cam1‐bound calcium (Fig. 2D). Displacement of Ca^2+^ from both forms of Cam1 occurred in two distinct phases of similar amplitude, indicating two classes of binding site, with two *k*_obs_ values that differed approximately 10‐fold (*k*_obs_ values 287 and 267 s^−1^ fast phase and 17.6 and 18.8 s^−1^ slow phase, for ^ACE^Cam1 and Cam1, respectively). However, the two amplitudes for ^ACE^Cam1 were significantly larger (Table 2).

**Table 2:** Quin2 rates and amplitudes.

	Rate 1 (s^−1^)	Ampl1	Rate 2 (s^−1^)	Ampl2	Rate 3 (s^−1^)	Ampl3
Cam1	267.66 (±7.55)	6	18.85 (±0.52)	7.2	3.43 (±0.10)	4.6
Nt‐acetylated Cam1	288.18 (±4.78)	15.7	17.61 (±0.11)	16.3		
hCaM	11.76 (±0.24)	17.8	4.58 (±0.14)	11.7		
Nt‐acetylated hCaM	9.31 (±0.02)	21.8				

Equivalent biochemical analyses were performed upon human calmodulin (Calmodulin‐1) protein. While we found no detectable differences in the structure, stability or conformation between the acetylated (^ACE^hCaM) and unacetylated (hCaM) proteins (not shown), as for Cam1, there were significant differences in the amount and rate of disassociation of Ca^2+^ between the two proteins (Fig. 2E, Table 2). Ca^2+^ displacement for ^ACE^hCaM appeared as a single exponential (*k*_obs_ – 9.3 s^−1^) similar to the slow phase for Cam1. The Ca^2+^ displacement from unacetylated hCaM could be best described by two phases of similar amplitude (*k*_obs_ of 11.7 and 4.6 s^−1^) but the two *k*_obs_ values differ by less than a factor of three and so are not well defined by the fit. Thus, the two classes of binding sites do not appear to differ significantly for the human protein.

To further examine differences in the calcium sensitivity of Cam1 and ^ACE^Cam1, a modified Cam1‐T6C protein was isolated in both acetylated and unacetylated forms, and labelled with the IAANS fluorescent probe. This fluorescent label reports on the surrounding local environment, and can be used to detect calcium binding at the N‐terminus of Cam1 [11]. From the pCa curve plotted from IAANS fluorescence changes in both Cam1 and ^ACE^Cam1, a pCa_50_ value of calcium binding can be determined (Fig. 2F). For ^ACE^Cam1‐IAANS, the fitted pCa_50_ value of 6.54 is 0.5 pCa unit higher than for the unacetylated form – 6.03. Together, these data show that Nt‐acetylation impacts the Ca^2+^ binding capacity for both human and fission yeast calmodulins.

### N‐terminal acetylation affects the affinity of Cam1 binding to Myo1 *in vitro*

Calmodulin light chains bind to IQ motifs in the neck region of myosins, regulating their function [29]. The fission yeast Class I myosin, Myo1 contains an IQ motif neck region which binds Cam1 [10, 11]. We previously described a recombinant Myo1IQ^12^‐FRET protein consisting of a donor CyPet fluorophore and an acceptor YPet fluorophore separated by a linker region of the two Myo1 IQ motifs [11]. Unbound IQ motifs have a flexible, collapsed conformation which allows FRET between the two fluorophores. Once light chains are bound to the IQ motifs, the neck region is stabilised in an extended conformation [23, 30], reducing observed FRET. Using this reported protein, we have previously shown that two molecules of acetylated Cam1 associate with the Myo1^IQ12^‐FRET protein in a calcium‐dependent manner [11].

**Fig. 3:**
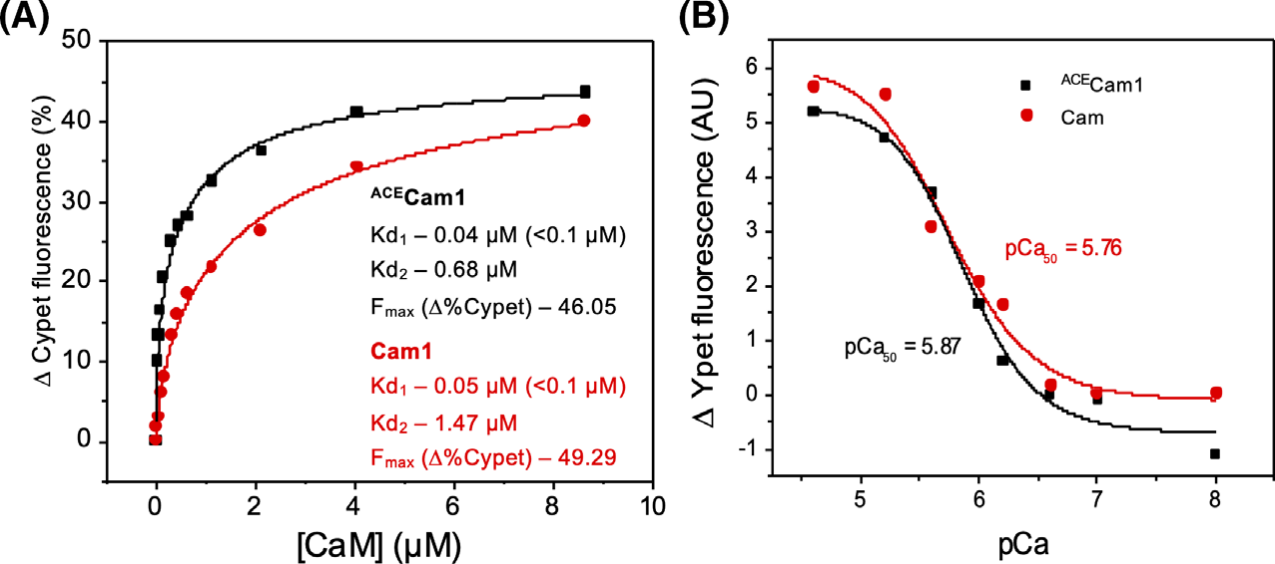
*In vitro* characterisation of Cam1 and ^ACE^Cam1. (A) Curves plotting Cam1‐dependent percentage changes in donor Cypet fluorescence signal of 0.5 µm of Myo1^IQ12^‐FRET proteins throughout a titration of Cam1 (red) or ^ACE^Cam1 (black). (B) pCa curves plotting Cam1 induced changes in Ypet fluorescence of 0.5 µm of Myo1^IQ12^‐FRET protein at a range of pCa conditions, with 0.8 µm of ^ACE^Cam1 (black) and 2.5 µm of Cam1 (red).

To determine the effect of Nt‐acetylation on the affinity of Cam1 for Myo1 IQ domains, Cam1 and ^ACE^Cam1 proteins were titrated into a 0.5 µm solution of Myo1^IQ12^ FRET protein in the absence of calcium. The % change in donor CyPet fluorescence was monitored to calculate changes in FRET caused by binding of Cam1 to the Myo1^IQ12^. Binding curves revealed that both Cam1 and ^ACE^Cam1 associate with the Myo1 IQ motifs, resulting in similar changes in CyPet signal (+46% and +49%, Fig. 3A). Analysis reveals both forms of Cam1 associate with the Myo1 IQ motifs in two distinct binding events. The first binding event, which accounts for ~ 50% of the total change in signal, corresponds to an affinity of < 0.1 µm for both Cam1 and ^ACE^Cam1, too tight to estimate with precision. However, the second weaker binding event differed significantly between Cam1 and ^ACE^Cam1. For ^ACE^Cam1, the affinity of this binding event was 0.68 µm, compared to 2‐fold weaker affinity of unacetylated Cam1 – 1.47 µm. This indicates that Nt‐acetylation of Cam1 increases the affinity for binding to Myo1 IQ domains, specifically affinity of the second molecule of Cam1.

### Nt‐acetylation does not affect the calcium dependency of the Cam1 interaction with Myo1

The interaction of Cam1 with the IQ motifs of Myo1 is tightly controlled by cellular calcium concentrations, when local or whole cell calcium concentrations rise, Cam1 dissociates from Myo1 [11, 31]. To explore whether Cam1 Nt‐acetylation affects this interaction, changes in FRET signal of the Myo1^IQ12^‐FRET protein induced by binding of half saturating concentrations of Cam1 (2.5 µm of Cam1, 0.80 µm of ^ACE^Cam1) were observed over a range of pCa conditions. The change in acceptor YPet fluorescence was used as a measure of FRET change induced by Cam1 binding, due to the changes in CyPet fluorescence being too small (Fig. 3B). There was no significant difference between the calculated pCa_50_ values for Cam1 – 5.76 and ^ACE^Cam1 – 5.87. Therefore, although Nt‐acetylation changes Cam1 affinity to Myo1 and sensitivity to calcium, it does not affect the calcium‐regulated interaction between Cam1 and Myo1 IQ domains.

## Discussion

Amino terminal acetylation is a ubiquitous post‐translational modification that affects the majority of eukaryote proteins [32]. Nt‐acetylation increases the overall propensity of the alpha‐helical structure within the fission yeast Cam1 protein (Fig. 2A), which is consistent with the α‐helical rich structure within the amino‐terminal calcium‐binding domains of CaMs [33, 34]. In addition, Nt‐acetylation enhances the overall stability of Cam1, as demonstrated by 100% of ^ACE^Cam1 refolding to its original helical‐structure after melting (Fig. 2B), which contrasts with equivalent unmodified Cam1, a significant proportion of which remains denatured after cooling. Although the melting temperature for ^ACE^Cam1 is lower than that of Cam1, this does not necessarily have a physiologically relevance for the protein.

While Cam1 and ^ACE^Cam1 both migrate through a gel filtration matrix at similar rates in the absence of calcium (Fig. 2C), indicating they have similar open structures, the addition of calcium only affected the progress of ^ACE^Cam1 through the matrix. This data suggests acetylation stabilises Cam1 structure to facilitate calcium‐binding associated changes to its conformation, which is consistent with the enhanced sensitivity to calcium observed in ^ACE^Cam1, compared to the unmodified protein (Fig. 2F). Interestingly the calcium dissociation rate constants (Table 2, rate 1 and rate 2) were equivalent for both proteins, indicating the ~ 3 fold difference in the calcium affinity ($K_{{\text{Ca}}^{2+}}=k_{\text{diss}}/k_{\text{assn}}$, Fig. 2F) for the two protein is a result of a change in the rate of calcium association (*k*_assn_). The difference in calcium affinities is part of the explanation for the difference in the amplitudes of the Quin‐2 fluorescence (Fig. 2D) the other being the smaller fraction of active, unacetylated CaM. Together, these data support a model in which Nt‐acetylation stabilises the alpha‐helical conformation of the EF domain containing amino terminal lobes of Cam1 to impact the affinity for Ca^2+^. Critically the Ca^2+^ binding capacity for both yeast and human CaM was regulated by Nt‐acetylation (Fig. 2D–F). Interestingly, acetylation has opposite effects upon Ca^2+^ release from the human and yeast calmodulins, which we are currently investigating the explanation for. However, this conserved biophysical property of calmodulins highlight the importance of ensuring recombinantly produced CaMs are Nt‐acetylated in order to ensure physiologically relevant data are obtained, which is particularly critical for developing calmodulin‐targeting therapies [35].

A significant proportion of cellular Cam1 associates with the sole fission yeast class I myosin, Myo1, in the cell [11]. Consistent with Nt‐acetylation enhancing Cam1 structure and calcium affinity, ^ACE^Cam1 had a 3‐fold tighter affinity for the Myo1 two IQ motifs compared to Cam1, which may indicate only 30% of the unmodified protein is folded correctly. This is also consistent with the observed differences in 222 nm alpha‐helix circular dichroism peaks (Fig. 2A). It is worth noting that the proportion of native yeast Cam1 that is acetylated on its amino‐terminus within the yeast cell (40%) [14], generating a subpopulation of CaM with distinct biophysical properties, coincides precisely with the proportion of discrete Cam1 foci (40%) that associates with Myo1 in the fission yeast cell [36]. It is interesting to speculate that the sub‐population of Cam1 stabilised by Nt‐acetylated is specifically tuned to regulate the function of specific proteins, including Myo1, in this cell.

The *naa15∆* deletion is likely to affect a wide range of cellular processes, as the NatA amino‐α‐acetyl‐transferase is responsible for the amino terminal acetylation of a significant proportion of eukaryote proteins (38% of human proteome) [14, 37]. However, we show that changes in Cam1 recruitment to sites of endocytosis and duration of the events is specifically due to amino‐terminal acetylation of the CaM as the *naa15∆* associated defects in Cam1 distribution and dynamics in the cell can be rescued by introducing an amino‐terminal GFP fusion to the protein. As the GFP‐Cam1 fusion cannot be amino‐terminally acetylated, we would not expect *naa15∆* to impact the distribution of GFP‐Cam1 in the cell. This is what was observed and illustrates the defects observed are specific to Cam1 and not secondary effect of acetylation of other proteins at the endocytic patch. Interestingly, differences in the position of the GFP label on Cam1 are reflected in differences in abilities to associate exclusively to either SPBs or dynamic endocytic foci. As the genes encoding for each fusion protein are expressed from the endogenous *cam1^+^* promoter at its chromosomal locus, this is likely to reflect differences in functionality. Here, we confirm that Cam1 binding to Myo1 impacts localisation of the motor to membrane at sites of endocytosis, affecting distribution and duration of the subsequent endocytic events [11]. Both unmodified and acetylated Cam1 bound to the two Myo1 IQ motifs (comparable amplitudes and 2 rates consistent with cooperative binding to the 2 IQ motifs – Fig. 3A).

Therefore, the reduced Cam1 signal at endocytic foci observed in *naa15∆* cells is likely to be due to fewer Myo1 molecules associating with the sites of endocytosis, rather than the unacetylated Cam1 only associating with a single Myo1 IQ motif. This may be due to the lower affinity of Myo1 for unmodified Cam1, which would bring about dissociation of the two proteins at lower concentrations of cellular calcium, despite the pCa of the interaction being unaffected by Cam1 acetylation, and therefore failure to associate with the membrane.

We show that amino‐terminal acetylation affects the conformation and calcium regulating function of calmodulins from fission yeast and humans, with a significant impact upon calcium affinity of each protein. These differences are not only of importance to researchers undertaking biochemical or structural studies of these conserved proteins, but should be considered when working upon recombinantly produced proteins, which are normally subject to this ubiquitous post‐translational modification(s) within their native cellular environment.

## Author contributions

Conceptualization: K.B., M.A.G., D.P.M.; Methodology: K.B., M.A.G., D.P.M.; Formal analysis: K.B., M.A.G., D.P.M.; Investigation: K.B., M.A.G., D.P.M.; Resources: M.A.G., D.P.M.; Data curation: D.P.M.; Writing ‐ original draft: M.A.G., D.P.M.; Writing ‐ review & editing: K.B., M.A.G., D.P.M.; Supervision: M.A.G., D.P.M.; Project administration: M.A.G., D.P.M.; Funding acquisition: D.P.M.

## Supporting information


**Fig. S1.** (A) Coomassie stained whole cell extracts from pre and post IPTG induction cell cultures with Cam1 co‐expressed with the NatA complex components Naa10 and Naa15. (B) Mass spectroscopy traces of purified bacterially expressed recombinant Cam1 and ^ACE^Cam1 protein. (C) Analysis of Cam1 foci distribution in *naa15^+^* (black circles) and *naa15∆* (red circles) cells.

## Data Availability

The data analysed and represented in this article are openly available and can be obtained from the University of Kent Data Repository (https://doi.org/10.22024/UniKent/01.01.417). Plasmid sequences and constructs from this study are deposited at addgene.org.

## References

[1] Chin D , Means AR . Calmodulin: a prototypical calcium sensor. Trends Cell Biol. 2000;10:322–8. 10884684 10.1016/s0962-8924(00)01800-6

[2] James P , Vorherr T , Carafoli E . Calmodulin‐binding domains: just two faced or multi‐faceted? Trends Biochem Sci. 1995;20:38–42. 7878743 10.1016/s0968-0004(00)88949-5

[3] Means AR , VanBerkum MF , Bagchi I , Lu KP , Rasmussen CD . Regulatory functions of calmodulin. Pharmacol Ther. 1991;50:255–70. 1763137 10.1016/0163-7258(91)90017-g

[4] O'Day DH . CaMBOT: profiling and characterizing calmodulin‐binding proteins. Cell Signal. 2003;15:347–54. 12618209 10.1016/s0898-6568(02)00116-x

[5] Heissler SM , Sellers JR . Various themes of myosin regulation. J Mol Biol. 2016;428:1927–46. 26827725 10.1016/j.jmb.2016.01.022 PMC4860093

[6] Adamek N , Coluccio LM , Geeves MA . Calcium sensitivity of the cross‐bridge cycle of Myo1c, the adaptation motor in the inner ear. Proc Natl Acad Sci USA. 2008;105:5710–5. 18391215 10.1073/pnas.0710520105 PMC2299219

[7] Moser MJ , Lee SY , Klevit RE , Davis TN . Ca2+ binding to calmodulin and its role in *Schizosaccharomyces pombe* as revealed by mutagenesis and NMR spectroscopy. J Biol Chem. 1995;270:20643–52. 7657644 10.1074/jbc.270.35.20643

[8] Moser MJ , Flory MR , Davis TN . Calmodulin localizes to the spindle pole body of *Schizosaccharomyces pombe* and performs an essential function in chromosome segregation. J Cell Sci. 1997;110(Pt 15):1805–12. 9264467 10.1242/jcs.110.15.1805

[9] Itadani A , Nakamura T , Hirata A , Shimoda C . *Schizosaccharomyces pombe* calmodulin, Cam1, plays a crucial role in sporulation by recruiting and stabilizing the spindle pole body components responsible for assembly of the forespore membrane. Eukaryot Cell. 2010;9:1925–35. 20833892 10.1128/EC.00022-10 PMC3008279

[10] Toya M , Motegi F , Nakano K , Mabuchi I , Yamamoto M . Identification and functional analysis of the gene for type I myosin in fission yeast. Genes Cells. 2001;6:187–99. 11260263 10.1046/j.1365-2443.2001.00414.x

[11] Baker K , Gyamfi IA , Mashanov GI , Molloy JE , Geeves MA , Mulvihill DP . TORC2‐Gad8‐dependent myosin phosphorylation modulates regulation by calcium. eLife. 2019;8:e51150. 31566560 10.7554/eLife.51150 PMC6802964

[12] Itadani A , Nakamura T , Shimoda C . Localization of type I myosin and F‐actin to the leading edge region of the forespore membrane in *Schizosaccharomyces pombe* . Cell Struct Funct. 2007;31:181–95. 10.1247/csf.06027 17202724

[13] Sammons MR , James ML , Clayton JE , Sladewski TE , Sirotkin V , Lord M . A calmodulin‐related light chain from fission yeast that functions with myosin‐I and PI 4‐kinase. J Cell Sci. 2011;124:2466–77. 21693583 10.1242/jcs.067850

[14] Arnesen T , Van Damme P , Polevoda B , Helsens K , Evjenth R , Colaert N , et al. Proteomics analyses reveal the evolutionary conservation and divergence of N‐terminal acetyltransferases from yeast and humans. Proc Natl Acad Sci USA. 2009;106:8157–62. 19420222 10.1073/pnas.0901931106 PMC2688859

[15] Starheim KK , Gevaert K , Arnesen T . Protein N‐terminal acetyltransferases: when the start matters. Trends Biochem Sci. 2012;37:152–61. 22405572 10.1016/j.tibs.2012.02.003

[16] Bähler J , Wu JQ , Longtine MS , Shah NG , McKenzie A , Steever AB , et al. Heterologous modules for efficient and versatile PCR‐based gene targeting in *Schizosaccharomyces pombe* . Yeast. 1998;14:943–51. 9717240 10.1002/(SICI)1097-0061(199807)14:10<943::AID-YEA292>3.0.CO;2-Y

[17] Eastwood TA , Baker K , Brooker HR , Frank S , Mulvihill DP . An enhanced recombinant amino‐terminal acetylation system and novel in vivo high‐throughput screen for molecules affecting α‐synuclein oligomerisation. FEBS Lett. 2017;106:8157–9. 10.1002/1873-3468.12597 PMC5396276 28214355

[18] Moreno S , Klar A , Nurse P . Molecular genetic analysis of fission yeast *Schizosaccharomyces pombe* . Methods Enzymol. 1991;194:795–823. 2005825 10.1016/0076-6879(91)94059-l

[19] Schmidt A , Kochanowski K , Vedelaar S , Ahrné E , Volkmer B , Callipo L , et al. The quantitative and condition‐dependent *Escherichia coli* proteome. Nat Biotechnol. 2016;34:104–10. 26641532 10.1038/nbt.3418 PMC4888949

[20] Rovere M , Powers AE , Patel DS , Bartels T . pTSara‐NatB, an improved N‐terminal acetylation system for recombinant protein expression in *E. coli* . PLoS One. 2018;13:e0198715. 29995905 10.1371/journal.pone.0198715 PMC6040700

[21] Carman PJ , Barrie KR , Dominguez R . Novel human cell expression method reveals the role and prevalence of posttranslational modification in nonmuscle tropomyosins. J Biol Chem. 2021;297:101154. 34478714 10.1016/j.jbc.2021.101154 PMC8463859

[22] Johnson M , Coulton AT , Geeves MA , Mulvihill DP . Targeted amino‐terminal acetylation of recombinant proteins in *E. coli* . PLoS One. 2010;5:e15801. 21203426 10.1371/journal.pone.0015801 PMC3009751

[23] Houdusse A , Gaucher J‐F , Krementsova E , Mui S , Trybus KM , Cohen C . Crystal structure of apo‐calmodulin bound to the first two IQ motifs of myosin V reveals essential recognition features. Proc Natl Acad Sci USA. 2006;103:19326–31. 17151196 10.1073/pnas.0609436103 PMC1687203

[24] Kelly SM , Price NC . The use of circular dichroism in the investigation of protein structure and function. Curr Protein Pept Sci. 2000;1:349–84. 12369905 10.2174/1389203003381315

[25] Bartels T , Kim NC , Luth ES , Selkoe DJ . N‐alpha‐acetylation of α‐synuclein increases its helical folding propensity, GM1 binding specificity and resistance to aggregation. PLoS One. 2014;9:e103727. 25075858 10.1371/journal.pone.0103727 PMC4116227

[26] Masino L , Martin SR , Bayley PM . Ligand binding and thermodynamic stability of a multidomain protein, calmodulin. Protein Sci. 2000;9:1519–29. 10975573 10.1110/ps.9.8.1519 PMC2144730

[27] Crivici A , Ikura M . Molecular and structural basis of target recognition by calmodulin. Annu Rev Biophys Biomol Struct. 1995;24:85–116. 7663132 10.1146/annurev.bb.24.060195.000505

[28] Tsien RY . New calcium indicators and buffers with high selectivity against magnesium and protons: design, synthesis, and properties of prototype structures. Biochemistry. 1980;19:2396–404. 6770893 10.1021/bi00552a018

[29] Heissler SM , Sellers JR . Kinetic adaptations of myosins for their diverse cellular functions. Traffic. 2016;17:839–59. 26929436 10.1111/tra.12388 PMC5067728

[30] Lu Q , Li J , Ye F , Zhang M . Structure of myosin‐1c tail bound to calmodulin provides insights into calcium‐mediated conformational coupling. Nat Struct Mol Biol. 2014;22:81–8. 25437912 10.1038/nsmb.2923

[31] Poddar A , Sidibe O , Ray A , Chen Q . Calcium spikes accompany cleavage furrow ingression and cell separation during fission yeast cytokinesis. Mol Biol Cell. 2021;32:15–27. 33175606 10.1091/mbc.E20-09-0609 PMC8098820

[32] Aksnes H , Ree R , Arnesen T . Co‐translational, post‐translational, and non‐catalytic roles of N‐terminal acetyltransferases. Mol Cell. 2019;73:1097–114. 30878283 10.1016/j.molcel.2019.02.007 PMC6962057

[33] Chattopadhyaya R , Meador WE , Means AR , Quiocho FA . Calmodulin structure refined at 1.7 A resolution. J Mol Biol. 1992;228:1177–92. 1474585 10.1016/0022-2836(92)90324-d

[34] Kuboniwa H , Tjandra N , Grzesiek S , Ren H , Klee CB , Bax A . Solution structure of calcium‐free calmodulin. Nat Struct Mol Biol. 1995;2:768–76. 10.1038/nsb0995-768 7552748

[35] Mayur YC , Jagadeesh S , Thimmaiah KN . Targeting calmodulin in reversing multi drug resistance in cancer cells. Mini Rev Med Chem. 2006;6:1383–9. 17168814 10.2174/138955706778993021

[36] Baker K , Kirkham S , Hálová L , Atkin J , Franz‐Wachtel M , Cobley D , et al. TOR complex 2 localises to the cytokinetic actomyosin ring and controls the fidelity of cytokinesis. J Cell Sci. 2016;129:2613–24. 27206859 10.1242/jcs.190124 PMC4958305

[37] Ree R , Varland S , Arnesen T . Spotlight on protein N‐terminal acetylation. Exp Mol Med. 2018;50:1–13. 10.1038/s12276-018-0116-z PMC6063853 30054468

